# Secretome of Stromal Cancer-Associated Fibroblasts (CAFs): Relevance in Cancer

**DOI:** 10.3390/cells12040628

**Published:** 2023-02-15

**Authors:** Deepshikha Mishra, Debabrata Banerjee

**Affiliations:** Department of Pharmacology, Robert Wood Johnson Medical School, Rutgers, The State University of New Jersey, Piscataway, NJ 08854, USA

**Keywords:** cancer, cancer associated fibroblasts, secretome, tumor microenvironment, stroma, tumor conditioned medium

## Abstract

The cancer secretome reflects the assortment of proteins released by cancer cells. Investigating cell secretomes not only provides a deeper knowledge of the healthy and transformed state but also helps in the discovery of novel biomarkers. Secretomes of cancer cells have been studied in the past, however, the secretome contribution of stromal cells needs to be studied. Cancer-associated fibroblasts (CAFs) are one of the predominantly present cell populations in the tumor microenvironment (TME). CAFs play key role in functions associated with matrix deposition and remodeling, reciprocal exchange of nutrients, and molecular interactions and signaling with neighboring cells in the TME. Investigating CAFs secretomes or CAFs-secreted factors would help in identifying novel CAF-specific biomarkers, unique druggable targets, and an improved understanding for personalized cancer diagnosis and prognosis. In this review, we have tried to include all studies available in PubMed with the keywords “CAFs Secretome”. We aim to provide a comprehensive summary of the studies investigating role of the CAF secretome on cancer development, progression, and therapeutic outcome. However, challenges associated with this process have also been addressed in the later sections. We have highlighted the functions and clinical relevance of secretome analysis in stromal CAF-rich cancer types. This review specifically discusses the secretome of stromal CAFs in cancers. A deeper understanding of the components of the CAF secretome and their interactions with cancer cells will help in the identification of personalized biomarkers and a more precise treatment plan.

## 1. Introduction

The term “secretome” was used originally by Tjalsma et al., in the context of proteins secreted by *Bacillus subtilis* [1]. In the human genome, secreted proteins represent a significant proportion of the total proteins encoded [2]. The secretome of a healthy human cell is comprised primarily of cytokines, growth factors, hormones, enzymes, glycoproteins, coagulation factors, and extracellular vesicles (EVs). EVs are a type of membrane-encapsulated particle that carry regulatory molecules, including RNA species (microRNAs, long non-coding RNAs, mRNAs), lipids, DNA fragments, oncoproteins, and oncopeptides from the donor to recipient cells. EVs include exosomes and micro-vesicles, help in cell–cell communication, and are of high significance in cancers [3]. A balanced secretome is vital for maintaining physiological homeostasis and a healthy condition. An in-depth investigation of cellular secretomes could potentially bring novel insights into the mechanisms behind disease development and eventually discover novel biomarkers and therapeutic targets. 

Performing a secretome profile of primary human cells from healthy donors can help in establishing a landscape of a healthy secretome that can be exploited for comprehensive human secretome data in the future. Such a human secretome atlas can be used as a reference for discovery of potential disease-associated biomarkers and eventually novel therapeutic targets [4]. Secretory proteins are also involved in the development of metabolic and neural diseases and may provide key knowledge about their pathological mechanisms [5]. A wealth of knowledge in the field of secretome biology comes from the studies investigating mesenchymal stem cells (MSCs). In the last decade, a large number of studies have investigated MSCs secretomes as a promising therapy for trauma and injuries [6]. Using the MSC secretome for treatment may help in overcoming hurdles such as oncogenic transformation, immunoreactivity, and high cost associated with conventional cellular therapy [7]. However, characterization of the components of the secretome is the first step towards biomarker identification and will require reliable, extremely sensitive assays [8]. 

As shown in Table 1, a total of 42 clinical studies appeared when searched for secretome data in ClinicalTrials.gov. (key word: “secretome”, data accessed on 9 February 2023). Thirty-six of studies found are represented in Table 1. A wide variety of clinical studies were found investigating the relevance and therapeutic potential of secretomes in various diseases, including cancers, COVID-19, obesity, Osteoarthritis, fertility, and more. Table 2. Summarizes secretome clinical data regarding cancers. Out of the 42, details of the remaining 6 studies are provided in Table 2. The clinical trial identifier number can be used to explore each study individually.

A deregulation in the constituents of the secretome is associated with development of the cancerous state [9]. The secretome regulates essential processes in cancers related to cell type differentiation, invasion, metastasis, and angiogenesis [10]. Tumor secretomes support the pro-tumorigenic processes via interleukins, including IL-6 and IL-8, members of the transforming growth factor (TGF) family, such as TGFβ, and secreted enzymes, such as matrix metalloproteinases (MMPs), capable of degrading the extracellular matrix (ECM), which together enable tumor cell migration and invasion. Glycoproteins such as clusterin, mucins, EV, and EV-derived miRNAs also help in this process [11]. The composition of the cancer secretome can be affected by alterations in de novo synthesis, half-life, and cellular trafficking of the secretome components. The secretome is also impacted by deregulations in the molecular signaling pathways. It can be suggested that altered cancer cell secretomes depend on multiple factors, although the processes that drive towards a more tumorigenic secretome are yet to be fully understood [12]. A secretome analysis allows the profiling and characterization of different soluble proteins, including cytokines and growth factors. 

When looking for clinical studies investigating cancer secretomes, we found six studies registered in ClinicalTrials.gov. (key word: “cancer secretome”, data accessed on 9 February 2023). The studies mentioned below are designed to explore the potential of the secretome in different cancers. Table 2 presents data regarding the recruitment status and identifier number for clinical trials exploring cancer secretomes.

**Figure 1:**
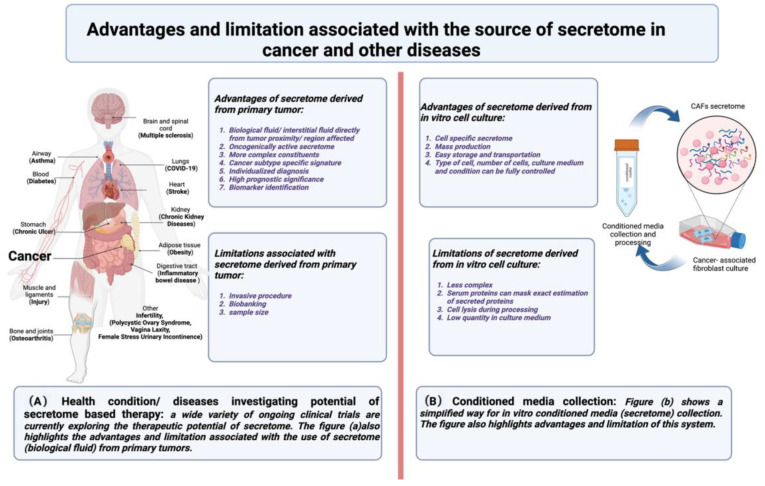
Secretome: Advantages and limitations; (**A**) shows different diseases exploring the clinical relevance of secretome-based therapy; (**B**) addresses the advantages and limitation associated with the use of CAFs (cell specific) secretomes in cancer.

The cancer secretome is also comprised of proteins released from the neighboring cancer-associated stromal cells, such as cancer-associated fibroblasts (CAFs) [13]. One of the broader methods of tumor– stroma communication is via paracrine interaction [14,15,16]. Cell conditioned media can be exploited as secretomes to study a variety of secreted factors playing a direct role in such interactions. Figure 1 provides a general view of the secretome and discusses pros and cons of the secretome as a source for future investigations and therapeutic designing. These secreted proteins are fundamental in cancer cell growth, proliferation, invasion, and angiogenesis by controlling cell–cell and cell–extracellular matrix interactions [17]. The following section summarizes the relevance of CAFs in the TME.

## 2. Carcinoma-Associated Fibroblasts

Carcinoma-associated fibroblasts are activated fibroblasts that represent one of the abundant cell populations present in tumors. CAFs contribute to cancer initiation and progression by delivering oncogenic signals in a paracrine manner [1]. CAFs are considered indispensable in the TME because they support tumor cells at every step of cancer progression by providing a structural and functional framework. CAFs produce chemokines, growth factors, and ECM structure that aids in the recruitment of other cell populations, such as endothelial cells and pericytes [18]. CAFs help in initiating metabolic and immune reprogramming that fosters a pro-tumorigenic microenvironment and may drive the tumor towards adaptive resistance to chemotherapy. CAFs display heterogeneity which imparts a context-dependent effect on cancer [19]. CAF heterogeneity can be attributed to various factors, including the fibroblasts they originate from, tissue position, morphology, and an absence of lineage markers for other cells. In a tumor biopsy, CAFs can be primarily identified by their elongated shapes and lack of markers for epithelial, endothelial, and leukocyte cells. Some of the most used markers for characterization include alpha-smooth muscle actin (αSMA), vimentin, fibroblast activation protein (FAP), and platelet-derived growth factor receptor α (PDGFRα) [20]. Often, negative markers such as epithelial cell adhesion molecule (EPCAM), caldesmon 1 (CALD1), and smoothelin (SMTN) are also used together in combination [21]. All abbreviations are also expanded at the end of the manuscript.

**Figure 2:**
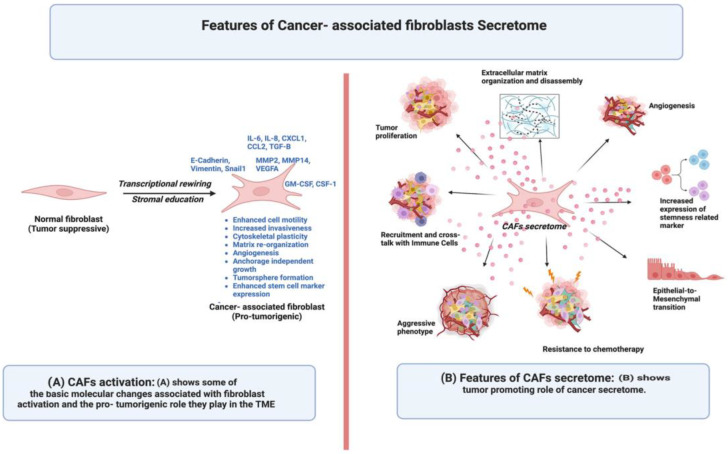
Features of CAFs secretome; (**A**) displays the process of fibroblast activation; (**B**) explains the diverse tumor-promoting features of the CAFs secretome.

As shown in Figure 2A, the transition of normal fibroblasts (NFs) to CAFs is a common phenomenon in the TME. It has been reported that p53 supported transcription is substantially altered in CAFs compared to NFs. This transcriptional rewiring renders an altered p53-dependent secretome in CAFs. It can be suggested that transcriptional rewiring mediates a non-mutational stromal education that aids the tumor suppressive to tumor supportive role of CAFs [22]. Molecular deregulations play a strong role in driving the process of activation of a normal fibroblast to CAF. Altered signaling pathways such as nuclear factor kappa B (NF-kB) signaling [23], p53 signaling [24], TGFβ family ligands signaling [25], and janus kinase 1 (JAK1) signaling [26] also help in the activation of fibroblasts via activating signal transduction.

Cancer-associated fibroblasts have gradually been viewed as key targets for therapeutic advancement in patients with cancer. Exploring CAF-specific features has been advantageous in the hunt for promising cancer biomarkers [27,28]. Sahai et al., have discussed a clinical trial targeting CAFs as clinically relevant targets. This is in view of the idea that exploring CAF-targeting strategies complementary to existing therapies would be of greater clinical benefit. For example, new small molecule inhibitors as anti-stromal drugs that can target crosstalk between CAFs and cancer cells are being investigated [29]. The following section explains features of the CAF secretome and its targeting.

## 3. CAF Secretome and Pharmacological Targeting 

An altered secretome shifts the balance towards substantial deregulation in proteins that promote the oncogenic processes (Figure 2B). The secretome of activated fibroblasts not only fosters differential regulation of protein expression, but the soluble factors present in the secretome stimulate the activity of enzymes such as metalloproteinases in the membrane of cancer cells, leading to the release of ligands that activate and upregulate associated signaling pathways. Additionally, the secretome of the activated fibroblasts also impacts the functional assembly of proteins. The secretome of activated fibroblasts may directly influence the process of protein homo- or hetero- dimerization to favor a highly oncogenic form [30].

Identification and characterization of CAF-derived proteins or the CAF secretome would help in understanding tumor stroma interactions in a way that would enable identification of CAF-specific molecular features that play a crucial role in cancer development. Investigating the CAFs secretome is not only crucial for understanding tumor behavior, but to also discover novel targets for cancer therapies. Investigating the role of the CAF secretome in different cancers is important for gaining novel insights about the tumor–stroma interactions. This section summarizes the data available regarding the CAF-derived secretome. Studies investigating CAFs secretome profiling and analysis were searched using the keywords: CAFs Secretome. The last study was accessed on 29 November 2022. 

Pharmacological targeting of stromal CAFs can be of clinical advantage in cancers with rich stroma with poor treatment response. Cancer-associated fibroblasts are often associated with drug resistance. Interestingly, CAFs’ effect on drug resistance is context dependent. Understanding the relationship between components of the CAF secretome and the signaling needs of different tumors may help in discovering effective combination treatment strategies. The tumor cell secretome of cancer cells is known to promote chemoresistance, tumor recurrence, and overall poor outcome. In this review, we have summarized studies investigating the CAF secretome and its relevance to cancer. Investigating stromal CAFs as potential therapeutics will help control a pro-tumorous secretome, thereby preventing CAF-mediated cancer progression. However, therapeutic targeting of the CAF secretome remains a challenge and the field and needs to be investigated in detail. The subsections summarize the data available regarding the secretome derived from cancer-specific CAFs and how they impact cancer development, progression, and treatment outcome.

### 3.1. Colorectal Carcinomas (CRC)

Worldwide, colorectal carcinoma ranks third among most diagnosed malignancies and stands second for being the leading cause of death. It is a heterogeneous disease involving different pathways in its development [31]. Colon cancer cells interact with stromal cells, immune cells, and the intestinal microbiome to drive progression of CRC [32]. CAFs play a crucial role in CRC progression and can predict poor prognosis in patients with CRC [33]. CAFs enhance tumor-initiating cells in CRC; transforming growth factor (TGF)-β signaling further increases it dramatically. It was observed that in all CRC subtypes with poor prognosis, stromal TGF-β played a significant role in tumor progression. TGF-β inhibitors blocked cancer cells’ interaction halting disease progression in patient-derived in vitro tumor models [34]. In mouse models of colitis-associated CRC, it was observed that stromal genes may contribute towards colorectal carcinogenesis. To investigate this further, a colitis-associated CRC mouse model was used. A lineage-tracing study was performed on FACS sorted CRC-CAFs. It was observed that ACTA2+ CAFs emerged from intestinal pericryptal leptin receptor (Lepr)+ cells through proliferation. Lepr-lineage CAFs expressed CRC stroma-specific marker melanoma cell adhesion molecule (MCAM), and a higher MCAM expression was inversely related to patient survival in CRC. In a mice model, stromal MCAM knockout blocked colorectal tumor growth and improved survival via decreasing the recruitment of tumor-associated macrophages. Fibroblast MCAM interaction with interleukin-1 receptor 1 augmented NF κB-IL34/CCL8 signaling that is responsible for macrophage chemotaxis. The MCAM+ CAFs leads to a pro-tumorigenic immune microenvironment [35].

Findings from CRC-CAF secretome analysis revealed a hybrid epithelial–mesenchymal transition (EMT) state mediated via hepatocyte growth factor (HGF) signaling. Fibroblasts derived from tumor region in CRC were found to express significantly elevated levels of CAF marker α-SMA [36]. Further, the secretomes of CAFs were seen to increase the expression of stemness-related markers including OCT4, CD44, CD133, and ALDH1A1 in human CRC Cells [37]. It is well established that paracrine signaling between CAFs and cancer cells drives a unique protein expression profile. In CRC, CAFs recruited via the growth factors derived from cancer cells display a myofibroblastic phenotype. To advance the understanding of colon cancer cell and CAFs interaction, proteomic analysis of conditioned media derived from a coculture model system has been performed. The proteomics data revealed increased expression of proteins associated with a myofibroblastic signature, including collagen type XII, collagen type III, and COL12A1 involved in regulating metastasis of colorectal cancer [38]. Comparative proteomic analysis of conditioned media from CAFs and NFs in colon cancer revealed an enrichment of proteins associated with the extracellular matrix, cell motion, adhesion, inflammatory response, peptidase inhibitor, and redox homeostasis in CM of CAFs. Proteins such as transgelin, decorin, and follistatin-related protein 1 (FSTL1) were present in abundance in the fibroblast secretome [39]. WNT2 was found to be specifically elevated in CRC CAFs, and the CRC-stromal-CAFs-derived WNT2 promoted angiogenesis by fostering pro-angiogenic signals. Mass spectrometry and cytokine arrays-mediated secretome profiling of CAFs revealed an elevation in proteins associated with pro-angiogenic functions, including IL-6, G-CSF, PGF, and ANG-2 [40]. Tenascin C, stromal-derived factor-1, and fibronectin ED-A domain have also been identified by differential secretome analysis in colon cancer [41].

### 3.2. Breast Cancer

Worldwide, breast cancer represents the most commonly occurring cancer in women, with a 70–80% cure rate in early-stage, non-metastatic patients. It is a heterogeneous disease [42]. Stromal CAFs present in breast cancer also display great heterogeneity. It has been observed that CAFs alter drug responses in breast cancer tumor cells, hence investigating the molecular mechanisms involved will likely help in identifying novel targets for improving therapeutic efficacy [43]. Commonly, breast cancer occurs in mammary ductal cells which produce nipple aspirate fluid (NAF). It was found through NAF secretome analysis in these cancer patients, that it was rich in proteins present in a tumor microenvironment. Using a paired-proteomic shotgun method for analysis of NAF from both breasts revealed an abundance glycolysis/Warburg effect-associated protein. Proteins involved in generating an activated stroma via immune system activation were also found to be significantly enriched. An abundance of proteins involved in promoting a proliferative microenvironment, such as the insulin-like growth factor, was also observed [44].

CAF-rich tumor stroma is associated with therapy resistance and inferior prognosis in breast cancer. Hence, it is of great interest to identify stroma-specific novel biomarkers. Experiments have been conducted on conditioned media mimicking tumor stroma interactions for this purpose. Constituents of CAF-conditioned medium were investigated to identify factors that may promote drug resistance in breast cancer cells. Secretome analysis of CAFs-CM exposed to HER2-positive metastatic breast cancer was performed. It was observed that factors from CAF-CM displayed the ability to promote resistance to first-line therapy, trastuzumab with pertuzumab and docetaxel. It was found that CAFs promoted a resistant phenotype in cancer cells via inducing EMT. It was observed that expression of mesenchymal markers, fibronectin and snail, were upregulated in breast cancer cells exposed to CAF-conditioned media [45]. Comparing the secretome of normal mammary cells and CAFs revealed an abundance of chloride intracellular channel protein 3 (CLIC3). It was seen that secreted CLIC3 supported invasive behavior of endothelial cells to drive increased angiogenesis and invasiveness of cancer cells. CLIC3 was also found to be abundantly present in both tumor and stromal regions of aggressive ovarian cancers that correlated with poor clinical outcomes [46].

### 3.3. Penile Carcinomas (PeCa)

Penile cancer comes under a rare type of genitourinary malignancy which is associated with poor disease outcome and limited therapeutic options. It is of high importance to understand the molecular interactions within the complex TME of penile tumors. Molecular profiling and characterization of deregulated genes and pathways will help in understanding the tumor/tumor microenvironment landscape and will develop novel treatment strategies [47]. Secretome analysis in PeCa revealed inflammation and extensive extracellular matrix remodeling. To understand the cellular complexity of stroma in PeCa, the secretory profile was investigated. Primarily upregulated secretory factors included collagens and MMPs. Clinical correlation studies revealed an association between higher proportion of CAFs and lower survival of patients. Patients with tumors showing greater CAF involvement showed a reduced proportion of immune cells, suggesting a possible crosstalk between CAFs and immune cells. Interestingly, patients with high CAF scores had elevated expression of MMPs and displayed an overall poor survival rate. An in vitro exposure of PeCa-derived CAFs with the MMP inhibitor GM6001 decreased cell viability significantly in penile CAFs compared to normal fibroblasts. This suggests that the use of MMP inhibitor GM6001 is of therapeutic relevance in CAFs-populated PeCa [48].

### 3.4. Head and Neck Squamous Cell Carcinomas (HNSCC)

Head and neck squamous cell carcinomas are an aggressive and heterogeneous type of tumor affecting millions of patients worldwide. Despite significant advances in therapeutics over the last several years, the 5-year survival rate has not improved beyond 50%. The involvement of different cells and cell–cell interactions in the complex TME has been implicated in tumor progression and therapy response [49]. Secretome analysis in HNSCC revealed the role of secretory factors secreted by the CAFs but not NFs, in promoting increased anchorage-independent growth, tumorsphere formation, and cancer stem cell marker expression, even in the absence of serum/supplements. The cytokines/paracrine factors secreted differentially by the CAFs and NFs were identified as modulators of epidermal growth factor receptor (EGFR), platelet-derived growth factor receptor (PDGFR), and insulin-like growth factor receptor (IGFR) activity. Inhibiting EGFR, IGFR, and PDGFR via pharmacologic targeting led to a significantly reduced CAF-induced anchorage-independent growth and tumorsphere formation, suggesting a crucial role of receptor tyrosine kinases including EGFR, PDGFR, and IGFR in supporting the CSC phenotype [50].

### 3.5. HepatoCellular Carcinoma (HCC)

CAFs play critical role in the TME of HCC. CAF-derived factors such as cardiotrophin-like cytokine factor 1 (CLCF1) increased expression of TGF-β and chemokine ligand 6 (CXCL6) secretion from tumor cells. The increased expression further stimulated stemness related features in the tumor cell in an autocrine fashion and increased infiltration of tumor-associated neutrophils (TANs) in a paracrine manner. The CLCF1-CXCL6/TGF-β axis plays a significant role in this cytokine-mediated cellular crosstalk that regulates CAFs, HCC progression, and patient prognosis [51]. Investigating a CAF-based risk signature is effective in predicting HCC prognosis [52]. In HCC-CAF, signature characterization revealed correlation with poor clinicopathologic features, along with liver cirrhosis, prominent EMT-associated markers, and overall poor survival. Co-culture with HCC CAFs induced EMT in HCC cells. A secretomic analysis revealed IL-6 and HGF as the main EMT-stimulating cytokines secreted by the HCC-CAFs [53]. Secretome analysis of the conditional medium (CM) of cultured CAFs derived from HCC specimens indicated deregulations in TGFβ-regulated secreted proteins, including semaphorin 7A (SEM7A), complement C1q tumor necrosis factor-related protein 3 (C1QT3), protocadherin gamma subfamily (PCDGK), glycoprotein nmb (GPNMB), and myoferlin (MYOF) [54].

### 3.6. Gastric Cancer (GC)

Colonization of Helicobacter pylori (Hp) initiates the chain of pathologic events in the gastric mucosa, including local inflammation, followed by gastric ulceration, and development of adenocarcinoma. Infection may also directly and indirectly affect the secretome of the tumor and CAFs. In vitro studies have been carried out to advance understanding regarding the interactions between gastric cells and Hp. Exposing gastric fibroblasts to Hp infection (cagA+vacA+) and studying their impact on normal epithelial cells revealed that conditioned media from Hp-activated gastric fibroblasts instigated EMT-like phenotypic transition and transmigration potential of normal epithelial cells. Gastric fibroblasts are one of the key targets of Hp infection and they communicate via paracrine interactions with epithelial cells and Hp. Hp-activated gastric fibroblasts differentiate toward a CAF-like phenotype, encouraging the EMT-related phenotypic changes in normal gastric epithelial cells [55]. Further, an altered NFκB and STAT3 signaling, along with Snail1, may help in defining a secretome that drives the fibroblast transition. This transition promotes a milieu that may fulfill one of the prerequisites for GC development [56]. Further, in GC, chemoresistance has been corelated with Hp infections. Exposing normal gastric epithelial cells to an Hp-activated gastric fibroblast secretome induced EMT and enhanced cell motility, invasiveness, and cytoskeletal plasticity. Together, it can be suggested that Hp-infected fibroblasts’ secretome stimulates reprogramming and re-organization of gastric niches that provides cues for GC progression [57]. It was revealed by secretome and transcriptome analysis in GC that predominantly expressed secretory protein IL-6 was CAF-specific and clinical data suggested that IL-6 was linked with a poor response to chemotherapy, suggesting a possible role in chemoresistance [58]. Hypoxic CAFs in GC promote tumor progression. Conditioned media from hypoxic CAFs promoted GC cell migration in a HIF-1α-independent manner via reducing expression of COL4A2, a protein involved in inhibiting angiogenesis and tumor growth [59]. 

### 3.7. Pancreatic Ductal Adenocarcinoma (PDAC)

PDAC counts among one of the most stroma–rich cancers [60,61]. A secretome analysis of conditioned media from a PDAC patient-derived primary CAF culture revealed a controlled stromal activity, including inflammatory responses after SOM230 (a sst1 agonist) treatment. Sst1 is a G-protein-coupled somatostatin receptor expressed by PDAC-CAFs. SOM230 treatment led to a significant reduction in metastasis in PDAC-harboring mice. It also diminished colony-stimulating factor 1 (CSF-1) levels in tumor and plasma. Stromal CSF-1 levels are associated with the aggressive phenotype in PDAC patients [62]. In PDAC, CAF targeting with a novel somatostatin analog, SOM230 (Pasireotide), checks the chemoprotective nature of the CAF secretome via inhibiting protein synthesis in CAFs. It was observed in primary cultures of CAF derived from human PDAC resections that the CAF secretome was able to stimulate survival, invasive, and migration features of cancer cells. Similarly, the CAF secretome stimulated epithelial-to-mesenchymal transition in cancer cells. All these features were abolished after SOM230 treatment in CAFs, highlighting a therapeutic potential of pharmacological targeting of stromal CAFs via SOM230 in PDAC [63].

### 3.8. Oral Squamous Cell Carcinoma (OSCC)

CAFs are known to play a very significant role in OSCC tumorigenesis. OSCC is one of the commonest types of oral cavity tumors [64]. Secretome analysis of OSCC-derived CAFs revealed a significant upregulation of proteins associated with extracellular matrix organization/disassembly and collagen metabolism, including the fibronectin type III domain-containing 1 (FNDC1), stanniocalcin 2 (STC2), and serpin peptidase inhibitor type 1 (SERPINE1), compared to normal oral fibroblasts (NOF). This upregulation in FNDC1, SERPINE1, and STC2, levels was directly associated with TGF-β1-mediated NOFs to CAFs conversion. Type I collagen, a major constituent of connective tissue, was also associated with several upregulated biological processes. Type I collagen protein was found to be significantly associated with shorter survival in OSCC patients [65]. Additionally, secretion of ROS by CAFs promotes aggressiveness of tumors in thyroid cancer and the secretome of CAFs reveals diverse soluble factors synthesized and secreted by CAFs that foster tumor progression within the TME [66,67].

### 3.9. Oral Tongue Squamous Cell Carcinoma (OTSCC)

Like in many other cancers, in OTSCC, a bidirectional crosstalk between CAFs and cancer cells helps in fostering a niche facilitated by secreted factors and various extracellular vesicles. To fully understand this process, patient tumor resected CAFs, isolated and characterized with matched adjacent normal fibroblasts, were used for proteomics analysis. Protein microfibril associated protein 5 (MFAP5) of extracellular microfibrils was found to be enriched in CAF secretomes. Clinical data revealed an association between MFAP5 expression and patient survival, pointing to a role of MFAP5 protein in OTSCC progression [68].

### 3.10. Melanoma

CAFs generated using conditioned media from primary and metastatic melanoma cells displayed elevated motility. CAFs’ secretome analysis revealed enhanced secretion of lactate, upregulated expression of MMPs mediators, several cytokines, including IL6, IL8, CXCL1, CCL2, ICAM1, and proteins associated with angiogenesis, such as VEGFA and GM-CSF. Highly aggressive melanoma cells displayed more prominent effects on the CAFs phenotype in terms of enhanced motility, expressed as an elevated migration and invasion ratio, as well as higher area of digestion in MMP2 and MMP14 proteolytic activity compared to less aggressive melanoma cells [69]. 

### 3.11. Ovarian Cancer

EVs are known for their role as information mediators between tumor and stromal cells. In ovarian cancer, it was observed that EVs released from human ovarian cancer cells were able to activate normal fibroblasts. EVs derived from more aggressive and less aggressive ovarian cancer cells exposed to fibroblasts led to NF activation. The secretome of activated fibroblasts was able to modulate features including proliferation, motility, and invasion of tumor, as well as fibroblasts, and endothelial cells. The findings support the idea that cancer cells can modify fibroblast behavior by release of EVs [70]. 

### 3.12. Lung Cancer

CAFs are known to display great heterogeneity in lung cancer and their functional classification is related to clinical response in patients with targeted therapies [71]. CAFs are known to enhance the metastatic ability of lung cancer cells via IL-6/STAT3 signaling. Mechanistically, it was observed that a conditioned medium derived from cultured CAFs significantly enhanced the migration and invasion potential of lung cancer cells by secreting IL-6, regulating EMT-associated markers such as E-cadherin and vimentin expression. Further, the expression of metastasis-associated genes including MMP-2 and VEGF was also found to be altered [72]. Exposure to CAF-conditioned media or co-culture with CAFs induced drug sensitivity in lung cancer cells. Secretome analysis indicated a differential expression of insulin-like growth factors (IGFs) and IGF-binding proteins (IGFBPs) in CAFs and normal lung fibroblasts. Recombinant IGFBPs treatment led to drug sensitization in gefitinib-resistant lung cancer cells [73].

## 4. Cellular and Environmental Factors Influencing CAFs Secretome 

The secretome of CAFs represents the assortment of proteins secreted or shed by the cell. The functional and clinical relevance of the secretome of cell populations, including the cancer stem cell [74], adipocytes [75], and CAFs [76], have been investigated to develop effective personalized cancer treatments. A comprehensive knowledge of cell specific secretome features and their interactions with different cell populations in the TME will assist in designing improved strategies, aiding a superior personalized approach for tumor management and treatment of cancer [19]. Secretomes derived from CAFs can directly and indirectly regulate tumor progression in multiple ways. One of the critical ways is via crosstalk with immune cells such as cytotoxic T cells (CTLs), helper T cells (Th), tumor-infiltrating lymphocytes (TILs), regulatory T cells (Tregs), myeloid-derived suppressor cells (MDSCs), mast cells (MCs), monocyte-infiltrating cells (MICs), and natural killer cells (NKs) [77]. In the breast cancer TME, it was observed that a CAF-derived secretome including cytokines/chemokines [78,79] and growth factors [80], together with additional secretory components, such as mRNAs, miRs, and other proteins, can directly impact cancer progression via immune cell polarization, leading to a pro-tumoral, immunosuppressive status. Like many other cancers, prostate cancer is also rich in stromal prostate CAFs, which are crucial in promoting tumor progression via their immunosuppressive properties. It was demonstrated via secretome analysis that a novel polysaccharide, MPSSS derived from Lentinus edodes, was able to inhibit the immunosuppressive nature of prostate CAFs, however, the detailed mechanism via which the MPSSS-treated prostate CAFs secretome influences prostate cancer progression needs to be investigated further [81].

The tumor microenvironment is highly complex with heterotypic interactions, hypoxia, inflammation, and redox imbalance. The tumor secretome adds to this complexity [82]. The complexity of the TME allows the transformation of normal resident fibroblasts to activated CAFs with elevated levels of reactive oxygen species (ROS). They are metabolically distinct from normal fibroblasts and are master secretors of proteins with pro-tumorigenic features. Hypoxia and angiogenesis are key features of a tumor that may influence the CAFs secretome by modulating different cellular and environmental factors. In the TME, blood vessels are found embedded in the complex tumor stroma. It is well known that intra-tumoral hypoxia leads to formation of dysfunctional blood vessels, which provides for tumor metastasis and poor efficacy of therapeutic treatments [83]. Data suggest that hypoxic condition and CAFs interact in the TME. It was observed that expression and secretion of different immunosuppressive factors including TGF-β, VEGF, programmed death-ligand 1 (PD-L1), IL6, and IL10 were increased in hypoxia. It was also observed that the secretome of hypoxic CAF exerts a more profound effect on cell-mediated cytotoxicity compared to its normoxic counterpart.

Crosstalk between CAFs and hypoxia is a key determinant in the complex immunosuppressive TME [84]. Hypoxia induces distinct remodeling of the CAF proteome. It has been observed in mammary CAFs that hypoxia induces molecular alterations to regulate angiogenesis. It was found through CAF secretome analysis that hypoxic CAFs fostered abnormalities in blood vessel formation by altering secretion of different proteins. One such uncharacterized protein, NCBP2-AS2, was found to be a regulator of angiogenesis and was renamed HIAR (hypoxia-induced angiogenesis regulator). HIAR was found to be present in the most abundance in hypoxic CAFs. NCBP2-AS2 silencing reduced expression of pro-angiogenic proteins VEGFA and STC1, whereas anti-angiogenic factor COL4A2 was found to be increased after NCBP2-AS2 silencing. HIAR silencing in endothelial cells diminished the pro-angiogenic and pro-migratory roles of hypoxic CAFs via reducing VEGF/VEGFR downstream signaling. CAF-endothelial cell co-culture models indicate that hypoxic human mammary CAFs encouraged angiogenesis via increasing NCBP2-AS2. Exposing endothelial cells to conditioned media from CAFs after silencing NCBP2-AS2 resulted in significantly reduced sprouting compared to CM derived from control CAFs [85]. The above finding indicates functional relevance of hypoxia in stimulating molecular alterations in mammary CAFs.

## 5. Secretome Profiling and Challenges

Technological advancements made in recent years in the field of proteomic analysis have greatly facilitated investigating the secretome in cancers. Broadly, two different approaches are used for secretome analysis. These approaches can be either proteomics-based or can rely on genome-based computational prediction. Proteomic approaches are the core strength of secretome analysis and help biomarker discovery in cancers. Largely, for secretome studies, three different methods of proteomic technologies are used: gel-based, MS-based methods (mass spectrometry, gel-free), or SELDI-TOF-MS (surface-enhanced laser desorption/ionization time-of-flight mass spectrometry)-based [10]. All these processes have their advantages and limitations. The reader is encouraged to read Xue et al. [10] for a detailed explanation of all methods.

A comparative proteomic analysis is especially helpful in investigating differential expression of secreted protein from normal fibroblasts and CAFs. Performing 2-D PAGE (Two-dimensional gel electrophoresis) and MALDI-TOF (matrix-assisted laser desorption/ionization time-of-flight) mass spectrometry allows for identification of differentially expressed spots. These spots (proteins) play essential roles in tumor development and progression. One such protein identified in the nasopharyngeal carcinoma CAF supernatant was galectin-1 [86]. Using ultrafiltration centrifugation for extracting secreted proteins followed by secretomic analysis allows identification of different proteins that may play a critical pro-tumorigenic role in cancer via regulation of different metabolic processes. In recent years techniques such as hotgun proteomic label-free quantification are used to investigate the changes in the proteome and secretome of cancer cells [87]. Further, implementing an integrated meta-analysis of proteome and secretome data may accelerate the process of potential biomarker identification for different cancers, including the stromal CAF-rich cancer types. Performing meta-analysis on mass spectrometry-based secretome data and combining with other clinically relevant data from publicly available databases, including The Cancer Genome Atlas (TCGA), will help in determining the prognostic relevance of significantly secreted proteins. It will help in an advanced prediction of survival outcome in a cancer-specific manner [88].

An advanced multi-omics platform to identify the biomarkers capable of predicting clinicopathological relevance and improving individualized prognosis based on the secretomic profile is a step in the right direction. Secreto-transcriptomics is a multi-omics technique to find breast cancer subtypes-specific candidate markers of prognostic significance in their oncogenically secreted states. This technique uses label-free quantitative technology to identify proteins showing BC-subtype specific biomarkers. SeCEP, a subtype specific secretion pattern, pinpointed key genes including CDH1, DDB1, CD44, HSPA5, and HSP90B1 involved in an oncogenically active secretome. The SeCEP gene data allowed identification of novel liquid biopsy biomarkers for prediction of advanced individualized prognosis [89]. SILAC-based secretome analysis from pancreatic cancer revealed an alteration in proteins such as cathepsin D, perlecan (HSPG2), fibronectin receptor (integrin β1), CD9 antigen, and profilin 1 proteins [90].

The field of secretome analysis has gained traction in recent years because of various advancements in proteomic technology. Establishing a clear and reliable method for sample collection and preparation for secretome analysis is still challenging, however, the major limitation with CAFs secretome analysis is the heterogeneity in the CAF population itself. Multiple factors impacting the diversity, such as the origin of CAFs, activation state, and response to stress, may encourage functional heterogeneity [91]. Further, because of their ability to differentially affect secretion of immunomodulatory cytokines, chemokines, and other soluble factors, CAFs support recruitment of immune cells in the tumor microenvironment. All these factors further add to the heterogeneity and differentiation process [92].

The sample preparation for secretome analysis is especially challenging. To investigate the cancer secretome, biological fluids in the tumor proximity or the interstitial fluid in the tumor region should be studied to estimate the proteins present. Using conditioned media samples derived from cancer cell lines is more commonly used for discoveries related to the cancer secretome. However, it has been found that conditioned media derived from in vitro cultured cells is significantly less complex compared to their biological fluid counterparts [93]. Further, using conditioned media has more challenges, e.g., serum proteins present in the culture media can mask the exact estimation of secreted proteins present in the CM. Cell lysis during the culture processing also influences constituents of the conditioned media. Additionally, the secreted proteins are often present in very low quantities in the culture media, making precise quantitation difficult [13].

## 6. Conclusions

Investigating a CAFs-derived secretome offers advantages to establish a CAF-specific profile. Further, studying the CAFs secretome will help in identification of clinically relevant biomarkers for CAF-dense cancer types. Investigating a conditioned medium from cancer cells and CAFs allows the identification of the assortment of secreted proteins promoting cancer proliferation and metastasis. Some of these proteins may also be useful in designing serum-based tests for early and non-invasive detection of cancer. Secretome profiling and analysis will speed up the process of diagnosis, prognosis, and monitoring of cancer [93]. 

Studies based on secretome profiling and analysis will support an efficient and personalized cancer care by adding parameters to address tumor recurrence, chemoresistance, and overall disease prognosis. However, certain limitations regarding the standardization process for the formulation of cell-conditioned media, characterization of biologically active molecules in the conditioned media, and the mode of action need to be overcome. Taken together, a deeper understanding of underlying factors that may introduce variability in the composition of the CAF-derived secretome needs to be investigated further. 

Through this review, we have focused on the broad contribution of the CAFs secretome towards inducing a pro-tumorigenic, immunomodulatory environment capable of restoring aberrant cancer cell growth and progression. Direct application of the CAFs secretome in the cancer therapeutic arena holds great promise but still has a long way to go. Nonetheless, having an advanced knowledge of the CAFs secretome will help in better management of stromal CAFs-rich cancers. Using this information for designing a combination therapy is of high clinical interest and relevance in the future. 

## Figures and Tables

**Table 1:** Clinical studies investigating the safety and potential of secretomes in different health conditions and diseases.

S.No.	Condition or Disease	ClinicalTrials.gov Identifier	Recruitment Status
1	Polycystic Ovary Syndrome	NCT05279768	Recruiting
2	Ocular Surface Disease	NCT05204329	Not yet recruiting
3	COVID-19	NCT04753476	Recruiting
4	Osteoarthritis	NCT04223622	Recruiting
5	Bone Loss	NCT04980261	Recruiting
6	Knee Osteoarthritis	NCT05579665	Active, not recruiting
7	Skin Aging	NCT05508191	Active, not recruiting
8	COVID-19	NCT05122234	Completed
9	COVID-19	NCT05019287	Completed
10	Inflammatory Bowel Diseases	NCT04136587	Recruiting
11	Type 2 Diabetes Mellitus	NCT03341793	Unknown
12	Female Infertility	NCT03042364	Completed
13	Obesity, Morbid Type2 Diabetes	NCT03093298	Completed
14	Osteoarthritis	NCT04314661	Recruiting
15	Cushing Syndrome	NCT03229395	Completed
16	Vagina Laxity	NCT05710536	Recruiting
17	Blastocyst (implantome)	NCT00505115	Completed
18	Multiple System Atrophy, Parkinsonism	NCT04876326	Recruiting
19	Prosthetic Joint Infection	NCT04661345	Recruiting
20	Ischemic Stroke	NCT05008588	Recruiting
21	Keloid	NCT04326959	Not yet recruiting
22	Female Stress Urinary Incontinence	NCT02023502	Recruiting
23	Asthma	NCT05478824	Not yet recruiting
24	Ligament Injury	NCT04889963	Recruiting
25	Chronic Ulcer	NCT04134676	Completed
26	COVID-19	NCT04903132	Recruiting
27	COVID-19	NCT04602442	Unknown
28	COVID-19	NCT04491240	Completed
29	Osteoarthritis	NCT05211986	Recruiting
30	Asthma	NCT04883320	Recruiting
31	Fertility	NCT02010424	Completed
32	Chronic Kidney Diseases	NCT05155267	Recruiting
33	Hidradenitis Suppurativa	NCT05208099	Not yet recruiting
34	Pulmonary Arterial Hypertension	NCT03905083	Withdrawn
35	Obesity	NCT04687540	Completed
36	Multiple Sclerosis	NCT04294979	Unknown

**Table 2:** Clinical studies investigating safety and potential of secretome in cancers.

S.No.	Cancer	ClinicalTrials.gov Identifier	Recruitment Status
1	Nasopharyngeal Cancer	NCT05261750	Recruiting
2	Ovarian Cancer	NCT05047926	Recruiting
3	Pancreatic cancer	NCT03791073	Recruiting
4	Head and Neck Cancer	NCT04007081	Completed
5	Non-Small Cell Lung Cancer	NCT02852083	Unknown
6	Melanoma	NCT02310451	Recruiting

## Data Availability

Not applicable.

## Abbreviations

αSMA: α-Smooth muscle actin; 2-D PAGE: Two-dimensional gel electrophoresis; C1QT3: Complement C1q Tumor Necrosis Factor-Related Protein 3; CAFs: Cancer-associated fibroblasts; CALD1: Caldesmon 1; CDH1: E-cadherin; CSF-1: Colony-stimulating factor 1; CTLs: Cytotoxic T cells; DDB1: Damage-specific DNA binding protein 1; ECM: Extracellular matrix; EMT: Epithelial–Mesenchymal Transition; EPCAM: Epithelial cell adhesion molecule; FACS: Fluorescence-activated cell sorting; FAP: Fibroblast activation protein; FNDC1: Fibronectin type III domain-containing 1; GPNMB: Glycoprotein Nonmetastatic Melanoma Protein B; HIAR: Hypoxia-induced angiogenesis regulator; Hp: Helicobacter pylori; HSP90B1: heat shock protein 90 beta family member 1; HSPA5: Heat shock protein family A member 5; IGFBPs: IGF-binding proteins; IGFs: Insulin-like growth factors; MALDI-TOF: Matrix-assisted laser desorption/ionization time-of-flight; MCs: Mast cells; MDSCS: Myeloid-derived suppressor cells; MICs: Monocyte-infiltrating cells; MMPs: Matrix metalloproteinases; MS: Mass spectrometry; MYOF: Myoferlin; NFκB: Nuclear factor kappa B; NKs: Natural killer cells; PCDGK: Protocadherin Gamma Subfamily; PDGFRα: Platelet-derived growth factor receptor-α; PTPRC: Protein tyrosine phosphatase, receptor type, C; ROS: Reactive oxygen species; SeCEP: Subtype specific secretion expression pattern; SELDI-TOF: Surface-enhanced laser desorption/ionization time-of-flight; SEM7A: Semaphorin 7A; SERPINE1: Serpin peptidase inhibitor type 1; SILAC: Stable isotope labeling with amino acids; SMTN: Smoothelin; STAT3: Signal transducer and activator of transcription 3; STC2: Stanniocalcin 2; TCGA: The Cancer Genome Atlas; TGF: Transforming Growth Factor; Th: Helper T cell; TILs: Tumor-infiltrating lymphocytes; TME: Tumor microenvironment; Tregs: Regulatory T cells.
